# Cancer risks and trends between 1997 and 2018, and effects of restored immunity in people living with HIV: Results from the ANRS CO4 French hospital database on HIV

**DOI:** 10.1002/ijc.70253

**Published:** 2025-11-20

**Authors:** Sophie Grabar, Paula Lakrout, Valérie Potard, Aurélien Belot, Jacques Cadranel, Christine Jacomet, Christine Katlama, Esaïe Marshall, Olivier Lambotte, Romain Palich, Sylvie Ronot‐Bregigeon, Jean‐Philippe Spano, Anne‐Marie Bouvier, Alain Makinson, Dominique Costagliola

**Affiliations:** ^1^ Sorbonne Université INSERM, Institut Pierre Louis d'Epidémiologie et de Santé Publique, AP‐HP Paris France; ^2^ Sorbonne Université INSERM, Institut Pierre Louis d'Epidémiologie et de Santé Publique Paris France; ^3^ Inequalities in Cancer Outcomes Network (ICON), Department of Health Services Research and Policy, Faculty of Public Health and Policy London School of Hygiene & Tropical Medicine London UK; ^4^ AP‐HP, Hôpital Tenon, Service de Pneumologie et Oncologie Thoracique, GRCO4 Theranoscan Sorbonne Université Paris France; ^5^ Infectious Diseases Department Clermont‐Ferrand University Hospital Clermont‐Ferrand France; ^6^ AP‐HP, Hôpital Pitié Salpêtrière Service de Maladies Infectieuses et Tropicales Paris France; ^7^ Université Paris Saclay, AP‐HP, Service de Médecine Interne Immunologie Clinique Hôpital de Bicêtre, Inserm Le Kremlin Bicêtre France; ^8^ Infectious Diseases Department, Hôtel‐Dieu Hospital, AP‐HP Paris Cité University Paris France; ^9^ APHM, Hôpital Sainte‐Marguerite Aix‐Marseille Université Marseille France; ^10^ AP‐HP, Hôpital Pitié Salpêtrière Service d'oncologie Médicale Paris France; ^11^ Digestive Cancer Registry of Burgundy Dijon France; ^12^ French Network of Cancer Registries (FRANCIM) Dijon France; ^13^ Dijon University Hospital Dijon France; ^14^ INSERM UMR 1231, EPICAD Dijon France; ^15^ Université de Bourgogne INSERM UMR 1231, EPICAD Dijon France; ^16^ CHU Montpellier, Service de Maladies Infectieuse et tropicale Université de Montpellier Montpellier France

**Keywords:** cancer, cohort, general population, HIV/AIDS, malignancy

## Abstract

We assessed long‐term trends in cancer incidence among people with HIV (PWH) in France between 1997 and 2018, focusing on AIDS‐defining cancers (ADC) (Kaposi's sarcoma, non‐Hodgkin's lymphoma, and cervical cancer), three virus‐related non‐AIDS‐defining cancers (Hodgkin lymphoma, liver, and anal cancer), and four virus‐unrelated cancers (lung, colorectal, prostate, and breast cancer). Using data from the ANRS CO4‐French Hospital Database on HIV and cancer registries in the general population, we calculated age‐standardized incidence rates and standardized incidence ratios (SIRs) across four time periods. Special attention was given to PWH with controlled viral load and restored CD4 during 2008–2018. Among 154,733 individuals contributing nearly 2 million person‐years, 9572 cancers were diagnosed. Incidence rates of ADC and virus‐related non‐ADC declined over time but remained significantly higher than in the general population, with SIRs ranging from 3 to 420, even in recent years. Rates of prostate and colorectal cancers increased overtime, while breast cancer incidence remained stable. For these three cancers, the relative risk compared to the general population remained close to 1. In PWH with CD4 ≥ 500/mm^3^ for at least 2 years and with recent viral load ≤50 copies/mL, risks of virus‐related cancers (KS, NHL, HL, liver, and anal cancer) remained significantly higher relative to the general population, albeit to a lesser extent than in PWH overall, while risks of lung and cervical cancers were similar. Over 20 years, the incidence of all virus‐related cancers continued to fall but in the most recent period, the risks still remained higher than in the general population.

## Graphical abstract

What's new?

This large nation‐wide cohort study provides a comprehensive 20‐year analysis of cancer trends among people living with HIV in France, highlighting persistent excess risks for virus‐related cancers despite effective antiretroviral therapy and immune restoration. By comparing outcomes with those observed in the general population and focusing on individuals with controlled HIV, the study highlights that cancer risk reduction is still incomplete. The findings support the need for tailored cancer screening and prevention strategies in people living with HIV, even in the context of long‐term viral suppression.

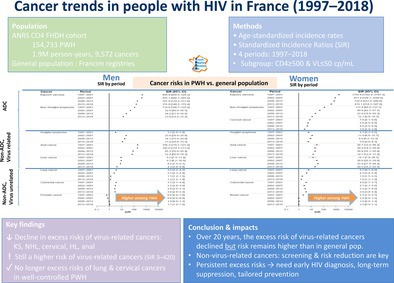

**Abbreviations**
ADC: AIDS‐defining cancers
ANRS CO4 FHDH: the French Hospital Database on HIV
cART: combination antiretroviral therapy
CNIL: Commission Nationale de l'Informatique et des Libertés
EGB: Echantillon généraliste des Bénéficiaires
FRANCIM: (France Cancer Incidence et Mortalité) French Network of Cancer Registries
HBV: hepatitis B virus
HCP: Healthcare Coverage Program
HCV: hepatitis C virus
HL: Hodgkin's lymphoma
ICD‐10: International Classification of Diseases, Tenth Revision
IQR: interquartile range
IR: incidence rate
KS: Kaposi's sarcoma
MSM: men who have sex with men
NADC: non AIDS‐defining cancers
NHL: non‐Hodgkin lymphoma
PWH: person with HIV
PY: person‐years
SIR: Standardized Incidence Ratio

## 1 INTRODUCTION

As HIV infection has progressively become a chronic condition with the advent in 1996 of combined antiretroviral therapy (cART), cancers have become a major cause of morbidity and mortality in ageing people with HIV infection (PWH).^1^ Over the past 20 years, a number of studies, including ours, have demonstrated an increased risk of cancers among PWH compared with the general population for both AIDS defining and some non‐AIDS defining cancers, with varying effects according to cancers.^2^, ^3^, ^4^, ^5^, ^6^ Some studies have also suggested a lower risk of breast, prostate, and colon cancer among PWH.^2^, ^5^, ^7^, ^8^ The increased risk of cancer in PWH is multifactorial, including effects of HIV replication, immunodeficiency, exposure to oncogenic viruses, and frequent exposures to toxic substances such as alcohol and tobacco in subsets of the population.

In Europe, northern America and Australia, incidence rates of most cancers decreased in PWH between 1996 and 2012.^4^, ^6^, ^9^, ^10^ However, these studies were conducted prior to the universal treatment of PWH regardless of CD4 cell count levels, recommended by WHO in 2016,^11^ and earlier in many high‐income countries such as France in 2013.^12^ In PWH with restored CD4 counts on cART, we previously showed that the risks of lung cancer and non‐Hodgkin's malignant lymphoma were no longer different from the general population,^6^, ^9^ but risks remained higher for other virus‐associated cancers. We were unable to estimate the risks for cervical cancer and anal cancer due to insufficient numbers.

With this in mind, we used data from the large French hospital database on HIV (ANRS‐CO4‐FHDH) cohort, and from the French epidemiological network of population‐based cancer registries (FRANCIM—France Cancer Incidence et Mortalité) to examine trends in the incidence of the three virus‐related AIDS‐defining cancers (ADC) (Kaposi's sarcoma (KS), non‐Hodgkin's lymphoma (NHL), and cervical cancer), three virus‐related non‐ADC (NADC) (Hodgkin lymphoma, liver and anal cancers), and four virus‐unrelated NADC (lung, colorectal, prostate, and breast cancers) among PWH relative to the general population between 1997 and 2018, also focusing on PWH on cART with controlled viral load and restored CD4 cell counts.

## 2 METHODS

### 2.1 Study population

We used data from the French Hospital Database on HIV (ANRS CO4 FHDH), an ongoing nationwide hospital‐based open cohort that includes data on PWH followed in 184 hospitals in 2020. To be enrolled in this cohort, participants must be at least 18 years of age, have a documented HIV‐1 or HIV‐2 infection, and have provided written informed consent.

This analysis included all PWH with HIV‐1 infection who were followed between 1 January 1997 and 31 December 2018 and aged between 18 and 84 years over this period. People with HIV‐2 infection were not included, as it is not known whether the risk of cancer is the same for people with HIV‐1 and people with HIV‐2. PWH from French overseas territories were not eligible as data on cancer in the general population were not available. As the analyses were done by sex and because exposure to hormones may influence the risk of cancer, transgender participants were excluded from the analyses. Participants were required to have at least one follow‐up visit (outpatient or hospitalization) or have died after the initial visit and have at least one CD4 and viral load measurement. For each calendar period, participants were included in the analysis if they had at least one visit during the period. Participants with previous cancers prior to the period considered were excluded from the analysis of the same cancer (Figure S1).

FRANCIM, which gathers data from 25 regional registries covering about 20% of the national population, provided incidence data in the general population for cancers diagnosed between 1997 and 2018, the latest data available.^13^ As there is no HIV status nor HCV/HBV status in French cancer registries, these incidences do not exclude cases occurring in PWH.

### 2.2 Statistical analysis

We studied the three virus‐related ADC (KS, NHL, and cervical cancer), three virus‐related NADC (Hodgkin lymphoma, liver, and anal cancers), and four virus‐unrelated NADC (lung, colorectal, prostate, and breast cancers). Invasive cervical and anal cancers reported in the FHDH were validated based on histological reviews retrieved from medical records. In FHDH, cancers are coded according to the International Classification of Diseases (ICD‐10), Tenth Revision. In FRANCIM, the new cases of cancers are registered using the International Classification of Diseases for Oncology 3rd edition (ICD‐O 3) (Table S2).

The 2006 ONCOVIH study revealed under‐reporting of cancer cases in the ANRS‐CO4‐FHDH, particularly for NADC.^14^ We thus corrected the number of cases of each type of cancer using different sources. For the 2008–2018 period, we estimated the notification rates from the permanent beneficiary sample (EGB), a representative sample of 1/97th of the population covered by health insurance schemes in France available since 2008. In the EGB, cancer diagnoses are provided by the French 100% Healthcare Coverage Program (HCP) for severe and costly long‐term disease, or as a primary or secondary cause of hospitalization. The notification rates were estimated by the ratios of the incidence rate (IR) of each cancer in ANRS CO4‐FHDH to the incidence rate in PWH in EGB. For 1997–2007, we estimated the notification rates from the 2006 ONCOVIH study.^14^ As colorectal and prostate cancers were not reported in the ONCOVIH study, the notification rates from EGB for the period 2008–2018 were used for these cancers for 1997–2007. The corrected number of cases for each period and cancer type was calculated by dividing the crude observed number of cases in the FHDH by the corresponding cancer's notification rate during that period. Given the small numbers of cancer cases in EGB, the same correction factor had to be used for all subgroup analyses (sex, age classes, HIV acquisition group or HBV/HCV infection status).

To explore the uncertainty of the notification rates, a first sensitivity analysis used the notification rates estimated from EGB for 2008–2018 for all periods. A second sensitivity analysis used the notification rates from ONCOVIH over 1997–2007 and those estimated from only the French 100% Healthcare Coverage Program (HCP) from 2008 (Table S3).

Similarly to previous works,^6^, ^9^ for each calendar period, the number of person‐years (PY) at risk for each patient was calculated from the date of cohort entry or the beginning of the period, whichever occurred later, until the date of cancer diagnosis, death, 6 months after the last visit, being older than 85 years of age, or the end of the period, whichever occurred first. We first estimated crude incidence rates over 1997–2007 and 2008–2018 by sex for each cancer. We then defined four calendar periods: 1997–2001, 2002–2007, 2008–2012 and 2013–2018 to assess temporal trends. To account for ageing of the PWH population and because of differences in age‐sex distributions between both populations, crude IR was then standardized for age (in 5 years increments) by using direct standardization based on the age distribution of the FHDH population followed between 2013 and 2018, separately for men and women. The analyses were conducted by sex and, for some cancers, by key populations (men who have sex with men, MSM), HBV or HCV coinfection or by smoking status. Standardized IR and their 95% confidence intervals (CI) in both populations were calculated for each period. To compare the risk of cancers in PWH to that of the general population, the standardized incidence ratios (SIR) (ratio of the observed number of cases in PWH with that expected in the general population) were used. The expected number of cases was estimated by weighting the age‐specific IR in men and women of the general population by the number of PY in the corresponding stratum of the PWH in each calendar period. The confidence intervals of the SIRs were calculated with an exact method based on the Poisson distribution. *p*‐values for trends for both IRs and SIRs were estimated with multivariable Poisson models with IRs or SIRs treated as the outcome and calendar periods as a covariate. In addition, to evaluate the risk of cancers in the subset of PWH with restored CD4 cell counts and controlled viral load, SIRs were estimated for participants followed between 2008 and 2018 whose CD4 count had been over 500 cells/mm^3^ continuously for at least 2 years and with controlled viral load <50 copies/mL at the last assessment before cancer or the last follow‐up. SAS software version 9.4 (SAS Institute, Cary, NC) was used for all statistical analyses.

## 3 RESULTS

A total of 154,733 PWH, contributing 1,909,585 person‐years of follow‐up were included in the study: 1,279,559 PY for men and 630,026 PY for women. The participants were followed in mainland France between 1997 and 2018 (Figure S1). Participants' characteristics are presented in Table 1. Median age increased from 36 years for the period 1997–2001 to 46 years in 2013–2018 for men and from 33 years to 42 years for women. Figure S2 shows the age structure of the PWH population in 2013–2018 and that of the general population in 2013–2018 and 2008–2012.

**TABLE 1:** Characteristics of person‐years contributed by the study population of PWH.

	Men								Women							
	1997–2001		2002–2007		2008–2012		2013–2018		1997–2001		2002–2007		2008–2012		2013–2018	
	PY		PY		PY		PY		PY		PY		PY		PY	
	203,459	%	297,814	%	317,735	%	460,551	%	83,200	%	145,157	%	167,737	%	233,932	%
Age (years)																
Median (q25–q75)^a^	36 (32–43)		39 (35–46)		43 (38–50)		46 (39–54)		33 (29–39)		36 (30–41)		39 (33–46)		42 (35–50)	
HIV acquisition group^a^																
Heterosexual	41,815	21	77,711	26	90,294	28	131,923	29	58,350	70	114,357	79	138,824	83	197,560	84
MSM	105,623	52	155,487	52	171,527	54	258,328	56	0	0	0	0	0	0	0	0
Injection drug users	41,960	21	44,523	15	35,486	11	39,612	9	16,506	20	17,432	12	14,310	9	15,598	7
Blood product	4100	2	4918	2	4340	1	5549	1	2853	4	4061	3	4269	3	5039	2
Missing or unknown	9961	5	15,175	5	16,088	5	25,139	5	5491	7	9306	6	10,334	6	15,736	7
Prior AIDS yes^a^	46,645	36	73,943	33	73,277	30	92,843	26	15,345	29	29,624	26	31,674	24	40,801	22
Origin^a^																
Sub‐Saharan	12,774	6	31,510	11	41,414	13	68,438	15	19,330	23	54,244	37	75,359	45	121,831	52
Others origin	190,685	94	266,304	89	276,321	87	392,113	85	63,871	77	90,913	63	92,378	55	112,102	48
Smoking status^a^																
Current					73,031	23	95,986	21					19,842	12	23,519	11
Former					78,576	27	119,686	28					35,655	23	56,137	25
Never					56,468	16	66,176	14					49,497	27	54,832	23
Missing or unknown					109,663	34	178,704	37					62,744	38	99,444	41
HBV or HCV hepatitis coinfection^a^	24,501	12	26,822	9	19,058	6	27,633	6	9152	11	11,616	8	8382	5	11,688	5
CD4																
≥500 cells/mm^3^	54,009	26	125,572	42	167,272	53	295,455	64	23,089	28	55,321	38	88,163	52	152,528	65
Median (q25–q75)^a^	444 (273–648)		461 (294–660)		483 (315–683)		496 (325–699)		466 (299–677)		476 (314–677)		491 (328–690)		500 (335–702)	
Viral load < 500 cp/mL	65,994	32	188,198	63	259,025	82	413,520	90	24,789	30	80,804	56	133,221	79	209,763	90
CD4/CD8 ratio ≥ 1	9837	5	26,490	8	56,139	17	138,296	30	6688	8	17,828	12	42,877	25	96,390	41
Median (q25–q75)^a^	0.49 (0.28–0.77)		0.52 (0.31–0.81)		0.55 (0.33–0.85)		0.57 (0.35–0.88)		0.60 (0.36–0.94)		0.63 (0.39–0.97)		0.66 (0 .41–1.0)		0.68 (0.42–1.03)	
Combined antiretroviral treatment	142,421	70	250,153	84	279,606	88	446,725	97	49,920	60	110,319	76	147,608	88	222,235	95

*Note*: A given patient may be followed up in more than one period.

Abbreviations: AIDS, acquired immunodeficiency; MSM, men having sex with men; PY, person‐years.

^a^ At entry of each calendar period.

Between 1997–2001 and 2013–2018, the proportion of person‐time of intravenous drug users decreased in both sexes. By contrast, it increased for sub‐Saharan Africa participants, rising from 23% to 52% among women, and from 6% to 15% among men. For HBV or HCV hepatitis co‐infected participants, proportions decreased from 12% to 6% for men and from 11% to 5% for women in the same periods. PWH with CD4 counts ≥500 cells/mm^3^ increased from 26% to 53% for men and 28% to 65% for women, PWH with viral loads <500 cp/mL increased from 32% to 90% for men and 30% to 90% for women, and PWH with CD4/CD8 ratio ≥1 increased from 5% to 30% for men and 8% to 41% for women.

### 3.1 Crude incidence rates

**FIGURE 1:**
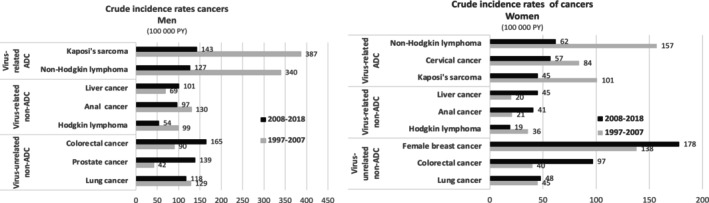
Crude incidence rates of cancer between 1997 and 2018. Corrected number of observed cases: The number of observed cases were corrected according to the estimated notification rates of cancer reporting in the ANRS CO4 FHDH obtained in the ONCOVIH study (Lanoy et al. Int J Cancer 2011) for the period 1997–2007 and using a representative sample of the French health insurance beneficiaries for the period 2008–2018 (Table S2). Person‐years by period: Men 1997–2007: 354,500 py – 2008–2018: 605,459 py. Women 1997–2007: 168,921 py – 2008–2018: 317,056 py. Median (Q25–Q75) age by sex and period: Men 1997–2007: 40 years (36–46) – 2008–2018: 43 years (36–50). Women 1997–2007: 37 years (32–42) – 2008–2018: 39 years (32–46).

Number of cancer cases and person‐years are given by sex, calendar period, and cancer type in Table S1. Figure 1 shows the evolution of the crude incidence rates of cancers across two decades. In both men and women, virus‐related ADC incidence rates fell sharply in 2008–2018. By contrast, incidence rates of virus‐unrelated ADC have risen considerably for colorectal, prostate and breast cancer, and were relatively stable for lung cancer. For virus‐related NADC, the incidence rates of HL fell in men and women, liver cancer increased in men and women, while for anal cancer it fell in men but increased in women.

### 3.2 Standardized incidence rates and standardized incidence ratios

**FIGURE 2:**
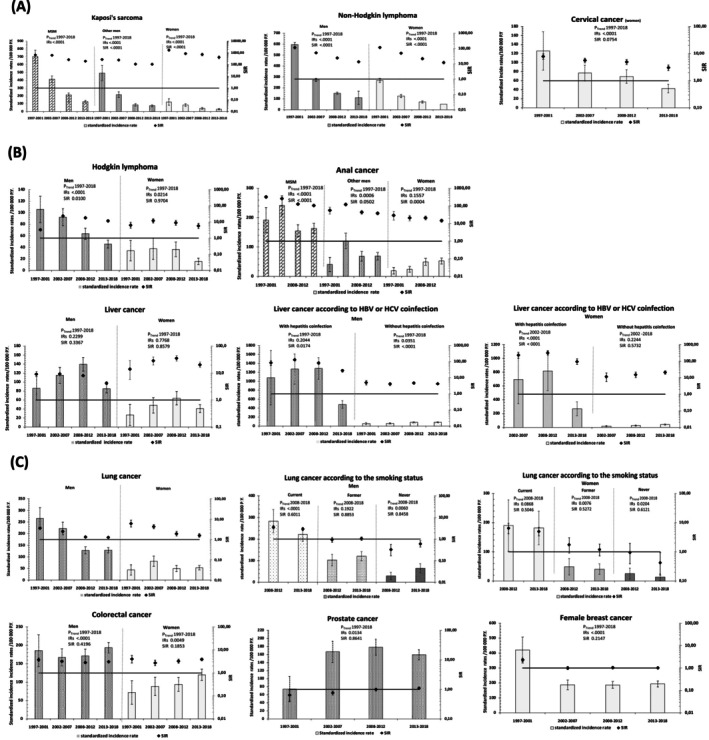
Age‐standardized incidence rates and standardized incidence ratios (SIR) with 95% confidence intervals in PWH over 1997–2018 by group, (A) Virus‐related AIDS‐defining cancers, (B) Virus‐related non‐AIDS‐defining cancers, (C) Virus‐unrelated non‐AIDS‐defining cancers. MSM, men who have sex with men; PY, person‐years; SIR, standardized incidence ratio. Incidence rates are standardized on the age distribution of PWH in FHDH between 2013 and 2018. Standardized incidence ratio (SIR) versus the general population. For liver cancer, the number of cases of HCV/HBV coinfected women, for the period 1997–2002 was too small to allow estimations. Smoking was recorded after 2005 in FHDH and was missing for 42% of PWH for patients followed since 2008.

Regarding virus‐related ADC (Figure 2A) both the standardized IR and the SIR fell significantly overtime for Kaposi's sarcoma and non‐Hodgkin lymphoma (*P*_trend_ < .0001); nonetheless, the SIR relative to the general population continued to be significantly elevated in the last period 2013–2018. For cervical cancer, the standardized IR fell too (*P*_trend_ < .0001); however, the SIR decreasing trends were not significant (*p*_trend_ = .07), being 3.0 (95% CI, 2.4–3.8) in 2013–2018 versus 7.8 (95% CI, 6.1–9.8) in 1997–2001. In sensitivity analyses, the same trends were observed except for cervical cancer, for which the decreasing trend in the SIR became significant when EGB notification rates were used for all the periods (Figure S3).

For virus‐related NADC (Figure 2B), Hodgkin lymphoma standardized IR and SIR fell significantly over time for men while for women only standardized IR declined significantly. For anal cancer, standardized IR and SIR decreased significantly only for MSM and other men but not for women. For liver cancer no trend could be evidenced overall for IR, but there was a significant decrease in SIR in men only. However, when limiting the analyses to participants living with HBV or HCV coinfection, SIR fell overtime for both men and women but remained significantly higher than in the general population at 27.6 (95% CI, 22.9–33.0) in men and 96.1 (95% CI, 63.3–139.9) in women in the last period. Similar trends were observed in the sensitivity analyses.

For virus‐unrelated NADC (Figure 2C), the lung cancer standardized IR fell in men but did not vary in women with no trend evidenced in SIR. When conducting the analyses according to smoking status over 2008–2018, lung cancer standardized IR fell in smoking men only, and no trend was evidenced in SIR in both sexes, at 3.0 (95% CI, 2.5–3.4) in men and 5.0 (95% CI, 3.5–6.9) in women in 2013–2018. There was an increasing trend in standardized IR for colorectal cancer in both men and women, but the SIR in both sexes did not change overtime and was significantly higher than in the general population at 3.0 (95% CI, 2.8–3.3) in men and 3.9 (95% CI, 3.4–4.4) in women in the last period. For prostate cancers, there was an increasing trend in standardized IR, but no significant change in SIR overtime, close to 1. Finally, for breast cancer in women, there was a decreasing trend in standardized IR, but no significant change in SIRs overtime, close to 1. Similar trends were observed in the sensitivity analyses (Figures S3 and S4).

**FIGURE 3:**
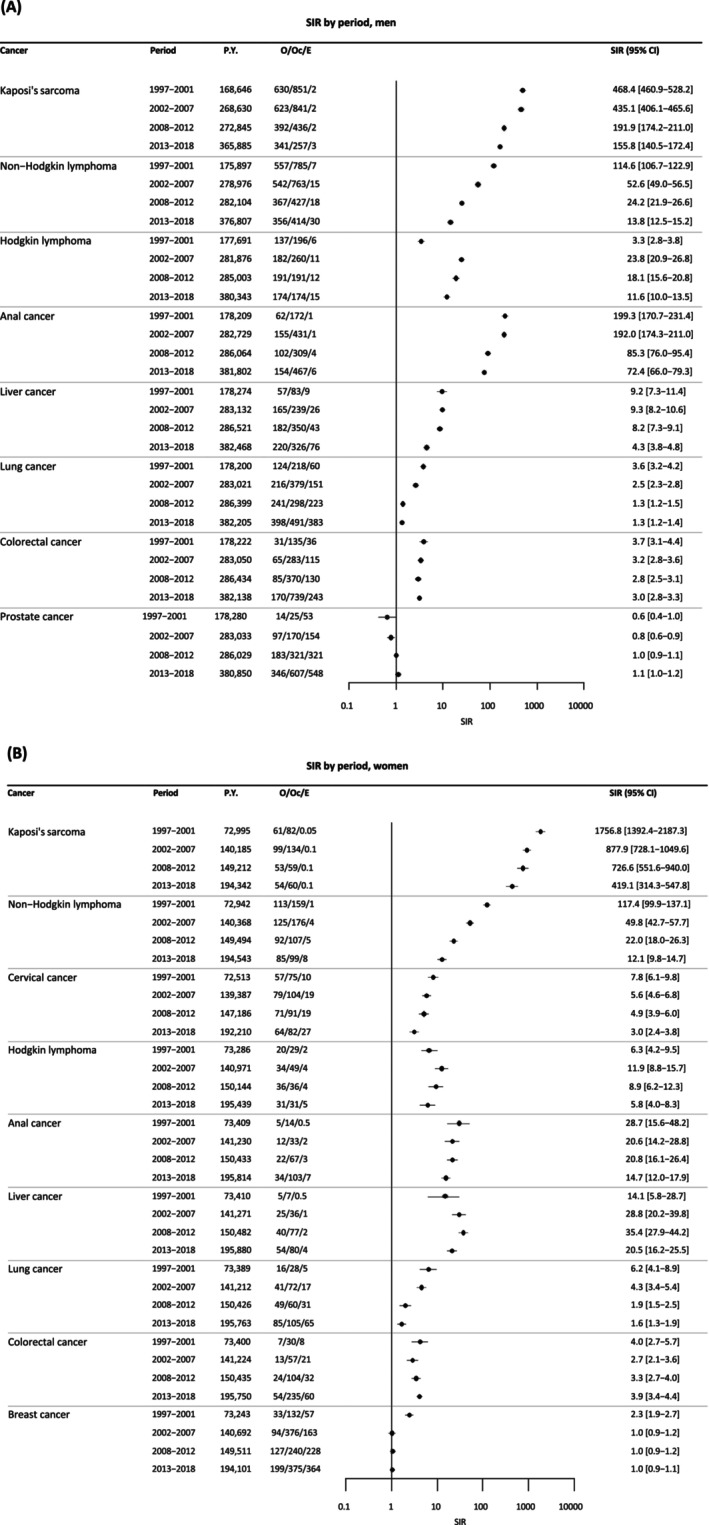
Trends of age‐standardized incidence ratios (SIR) of cancers in PWH over 1997–2018, by sex, (A) men, (B) women.

**FIGURE 4:**
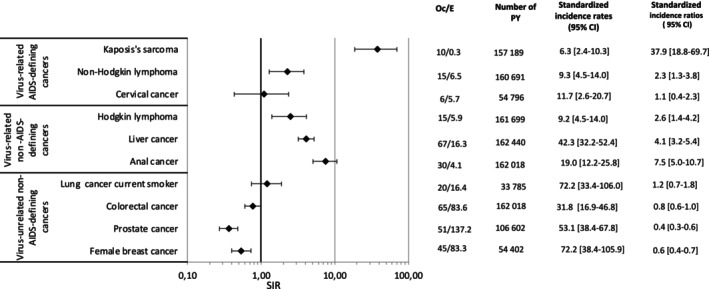
Age‐standardized incidence ratios (SIR) in PWH with CD4 ≥ 500/mm^3^ for at least two years and controlled viral load (<50 copies/mL) over 2008–2018. CI, confidence intervals; E, number of expected cases; Oc, corrected number of observed cases; SIR, Age‐standardized incidence ratio (SIR) in PWH versus the general population; PY, person‐years. Incidence rates are standardized on the age distribution of PWH in FHDH between 2013 and 2018 (see Figure S2).

Figure 3 shows SIRs by cancer and by calendar periods overall. In participants with CD4 > 500/mm^3^ and controlled viral load (<50 copies/mL) (Figure 4), the risk of cancer remained elevated relative to the general population for KS, NHL, HL, liver, and anal cancers, while the risk was no longer significant for lung, colorectal, and cervical cancer.

## 4 DISCUSSION

Using a large nationwide cohort, we examined trends in the incidence of virus‐related ADC, three virus‐related NADC, and four virus‐unrelated NADC among PWH relative to the general population from 1997 to 2018. Over 20 years, the incidence of all ADC and virus‐related NADC declined but remained higher than in the general population, even in the most recent period (2013–2018). The standardized IRs of prostate and colorectal cancer increased over time, but not the IRs of breast cancer. Relative to the general population, the risks of these three cancers remained stable and similar for prostate and breast cancers. In PWH with restored CD4 counts, the risk of most virus‐related cancers remained elevated but was lower than in PWH overall, while lung and cervical cancer risks were similar to the general population.

Strengths of our study include its large population size and duration, allowing risk estimations by cancer, sex, and period. Another strength was the review of histopathology reports for invasive anal and cervical cancer diagnoses, preventing misclassification. In our study, 40% of anal and cervical cancer cases in the FHDH were pre‐cancerous upon review. Cancer underreporting is a major concern in PWH cohorts without registry linkage.^9^, ^15^, ^16^ To correct under‐notification and in the absence of a national cancer registry in France, and of HIV status in existing French cancer registries, we used data from a sample of the French Health Insurance beneficiaries. Reporting rates were highest for AIDS‐defining cancers, Hodgkin's lymphoma, and lung cancer but lower for non‐AIDS‐defining cancers. However, the sample size was insufficient to allow corrections by sex, age, HIV acquisition group or period, and was only available from 2008, which justified the use of other sources for the correction and the fact that the correction factors were therefore applied in the same way according to age, sex, and HIV acquisition group. This may have biased risk estimates, but probably not trends over time. Sensitivity analyses performed with different correction factors confirmed our results. Estimating cancer incidence rates according to exposure factors such as tobacco and hepatitis C/B is also a strength of our study, though smoking status was unknown prior to 2006 and missing for 30%–40% of participants thereafter. Such analyses cannot be conducted in data linkage studies like in the US^17^, ^18^ or Australia^10^ due to a lack of smoking status information. Unfortunately, these exposure factors are not available in the general population registry data (Francim data). Therefore, the analyses by exposure group may have overestimated the SIRs in the “exposed groups” (smokers or HBV/HCV infected PWH) and underestimated the SIRs in the “non‐exposed groups” (non‐smokers or HBV/HCV uninfected PWH).

Our analysis reflects the changing cancer spectrum in PWH in high‐income settings. Our results align with projections from the HIV/AIDS Cancer Match Study, predicting prostate, lung, liver, and anal cancers as the most prevalent in the USA by 2030.^19^ Our findings confirm higher ADC and virus‐related NADC risks compared to the general population, though decreasing over time. Recently, experts recommended discontinuing the term ADC, considering it obsolete and incomplete to capture all cancers associated with HIV‐induced immunodeficiency and advised considering each cancer arising in PWH separately.^20^

The crude and age‐standardized reduction in incidence of KS, NHL, cervical cancer, and HL reflects the beneficial effect of cART and restored CD4 cell counts on virus‐associated cancers. SIR of these four cancers also declined, though not significantly for cervical cancer, probably underscoring the beneficial effect of improved general immunity associated with earlier HIV diagnosis and cART. Similar evolutions were described for HL in men. However, for all these cancers, IR was high and SIR remained increased relative to the general population in all periods. In France, a large proportion of HIV diagnosis is still at an advanced stage (CD4 < 200/mm^3^ and/or AIDS), estimated at 27% in France in 2023, a proportion stable since 2012, with a significant proportion of these cases with virus‐induced cancers.^21^ Another explanation is that despite cART, a significant proportion of PWH do not fully restore their CD4 levels and/or CD4/CD8 ratio above 1. This proportion can even be the majority, notably in those who started cART at low CD4 levels, more advanced age, or with AIDS‐associated infections. A low CD4/CD8 ratio has been shown to be associated with cancers in PWH,^22^, ^23^ mostly virus‐associated, and lung cancer.^24^ In addition, some cases of KS are now being observed in patients with high CD4 counts and undetectable HIV viral loads.^25^, ^26^ The physiopathology of these KS has yet to be explained, but loss of anti‐HHV8 immunological response, and the effect of immunosenescence have been suggested.^27^ These cases of KS in aviremic HIV cases may account for a proportion of the KS cases in PWH, particularly in the more recent periods.

SIR remained significantly higher relative to the general population even in PWH with controlled disease for all ADC, but non‐significantly for cervical cancer, despite decreasing trends with advancing calendar periods. These findings may reflect an increased risk of these cancers at all CD4 strata relative to the general population, though the stepwise decrease of risk in these cancers correlated with increasing CD4 immunodeficiency may explain the decreasing trend in SIR.^28^, ^29^ It would be interesting to evaluate IR, SIR of these cancers in PWH with CD4 > 500/mm^3^ and normal CD4/CD8 ratio, another marker of immune deficiency and activation, but our study would be underpowered for such an analysis.

Liver cancer crude incidence increased between 1997–2007 and 2008–2018, a probable effect of ageing. Age‐standardized IR was much higher in PWH with HBV/HBC coinfection than without and was reduced in the last calendar period (2013–2018), driven primarily by the strong reduction of liver cancer in HBV/HCV co‐infected PWH. Interestingly, the standardized IR of non‐viral related liver cancers increased significantly in men, which may reflect the metabolic‐associated epidemic of liver cancers in PWH as in the general population. In France, universal treatment of HCV in co‐infected PWH was recommended in 2014. Systematic treatment of co‐infected HBV in PWH with cART comprising tenofovir disoproxil fumarate was advocated by national recommendations in 2008. However, IR of these HCV/HBV liver cancer was still high, albeit decreasing in the last calendar period. This reflects that HCV/HBV treatment has not yet annihilated the risk of liver cancer, nor of its associated mortality.^30^, ^31^

SIR reductions for anal cancer in MSM and cervical cancer in women with HIV may reflect improved immunity and screening programs. Annual anal cancer screening for MSM PWH has been recommended in France since 2013. A Dutch study showed declining anal cancer IR in MSM but increasing rates in other groups without screening.^32^ New French guidelines recommend screening for women with HIV and a history of cervical or vulvar cancer, factors associated with anal cancer risk.^33^, ^34^

Our findings highlight the role of exposure factors in lung and other cancers. Smoking is the primary cause of lung cancer, reflected in our study by IR differences between never, former, and current smokers. Reduced smoking exposure may partially explain lung cancer incidence declines overtime. Similar IRs between PWH with controlled HIV disease and the general population suggest moderate immunodeficiency effects on lung cancer risk, mostly at low CD4 levels.^28^ However, CD4 recovery in recent periods also likely contributed to decreasing lung cancer incidence.

The increasing crude incidence of prostate, breast, and colorectal cancers reflects ageing and the importance of standard screening. Two meta‐analyses found similar IRs in PWH, transplant patients, and the general population, suggesting no immunodeficiency effect on these cancers.^2^, ^3^ The lower SIRs for prostate and breast cancer in PWH with controlled disease require further investigation. Cancer stage at diagnosis could clarify whether lower screening compliance contributes to these findings.

## 5 CONCLUSION

Over the past two decades, the incidence of many virus‐related cancers has declined among PWH, yet the risk remains significantly higher than in the general population. For non‐virus‐related cancers, controlling risk factors such as smoking and following general cancer screening guidelines is crucial. In PWH with stably restored CD4 cell counts, the persistent elevated risk of virus‐related cancers highlights the importance of early HIV diagnosis and treatment to prevent severe immunodeficiency and reduce cancer burden. Future research should focus on refining cancer risk estimates and optimizing prevention strategies tailored to PWH.

## AUTHOR CONTRIBUTIONS

**Sophie Grabar:** Conceptualization; funding acquisition; writing – original draft; methodology; validation; supervision. **Paula Lakrout:** Formal analysis; data curation. **Valérie Potard:** Formal analysis. **Aurélien Belot:** Writing – review and editing. **Jacques Cadranel:** Writing – review and editing. **Christine Jacomet:** Writing – review and editing. **Christine Katlama:** Writing – review and editing. **Esaïe Marshall:** Writing – review and editing; data curation; investigation. **Olivier Lambotte:** Writing – review and editing. **Romain Palich:** Writing – review and editing. **Sylvie Ronot‐Bregigeon:** Writing – review and editing. **Jean‐Philippe Spano:** Writing – review and editing. **Anne‐Marie Bouvier:** Writing – review and editing. **Alain Makinson:** Writing – review and editing; writing – original draft. **Dominique Costagliola:** Conceptualization; methodology; writing – original draft; writing – review and editing.

## FUNDING INFORMATION

The ANRS CO4 FHDH is supported by the ANRS‐MIE (Agence Nationale de Recherche sur le Sida et les hépatites virales‐Maladies Infectieuses Emergentes), INSERM (Institut National de la Santé et de la Recherche Médicale) and the French Ministry of Health. This work received the grant from ANRS‐MIE: ANRS‐MIE AAP 2020‐1 (project n°ECTZ115869).

## CONFLICT OF INTEREST STATEMENT

JPS received a research grant from MSD Avenir, honoraria from MSD and Incyte as a consultant. He also received honoraria from Pfizer, Lilly, Gilead, Novartis, PFO as a symposium presenter and participation in the advisory board. RP has received travel support and honoraria from ViiV Healthcare, Gilead and MSD. The other authors declare no conflict of interest.

## ETHICS STATEMENT

The ANRS CO4 FHDH cohort was initially approved by the Commission Nationale de l'Informatique et des Libertés (CNIL) on November 27, 1991. Renewed authorizations were obtained on February 19 and March 30, 2021 from the CNIL. All participants included in ANRS CO4 FHDH have provided written informed consent.

## Supporting information


**Appendix S1:** Supporting information.

## Data Availability

The data that support the findings of this study are available from the corresponding author upon reasonable request and access to the data on a secure platform can be granted following approval from the French data protection agency (CNIL).

## References

[1] Trickey A , McGinnis K , Gill MJ , et al. Longitudinal trends in causes of death among adults with HIV on antiretroviral therapy in Europe and North America from 1996 to 2020: a collaboration of cohort studies. Lancet HIV. 2024;11(3):e176‐e185. doi:10.1016/S2352-3018(23)00272-2 38280393 PMC11656032

[2] Jin F , Vajdic CM , Poynten IM , McGee‐Avila JK , Castle PE , Grulich AE . Cancer risk in people living with HIV and solid organ transplant recipients: a systematic review and meta‐analysis. Lancet Oncol. 2024;25(Jul):933‐944. doi:10.1016/S1470-2045(24)00189-X 38936380 PMC11246791

[3] Yuan T , Hu Y , Zhou X , et al. Incidence and mortality of non‐AIDS‐defining cancers among people living with HIV: a systematic review and meta‐analysis. EClinical Medicine. 2022;52:101613. doi:10.1016/j.eclinm.2022.101613 PMC9386399 35990580

[4] Hernandez‐Ramirez RU , Shiels MS , Dubrow R , Engels EA . Cancer risk in HIV‐infected people in the USA from 1996 to 2012: a population‐based, registry‐linkage study. Lancet HIV. 2017;4(11):e495‐e504. doi:10.1016/S2352-3018(17)30125-X 28803888 PMC5669995

[5] Hessol NA , Whittemore H , Vittinghoff E , et al. Incidence of first and second primary cancers diagnosed among people with HIV, 1985–2013: a population‐based, registry linkage study. Lancet HIV. 2018;5(11):e647‐e655. doi:10.1016/S2352-3018(18)30179-6 30245004

[6] Hleyhel M , Belot A , Bouvier AM , et al. Risk of AIDS‐defining cancers among HIV‐1‐infected patients in France between 1992 and 2009: results from the FHDH‐ANRS CO4 cohort. Clin Infect Dis. 2013;57(11):1638‐1647. doi:10.1093/cid/cit497 23899679

[7] Coghill AE , Engels EA , Schymura MJ , Mahale P , Shiels MS . Risk of breast, prostate, and colorectal cancer diagnoses among HIV‐infected individuals in the United States. J Natl Cancer Inst. 2018;110(9):959‐966. doi:10.1093/jnci/djy010 29529223 PMC6136931

[8] Marcus JL , Chao CR , Leyden WA , et al. Prostate cancer incidence and prostate‐specific antigen testing among HIV‐positive and HIV‐negative men. J Acquir Immune Defic Syndr. 2014;66(5):495‐502. doi:10.1097/QAI.0000000000000202 24820107

[9] Hleyhel M , Bouvier AM , Belot A , et al. Risk of non‐AIDS‐defining cancers among HIV‐1‐infected individuals in France between 1997 and 2009: results from a French cohort. Aids. 2014;28(14):2109‐2118. doi:10.1097/QAD.0000000000000382 25265077

[10] Wong IKJ , Grulich AE , Poynten IM , et al. Time trends in cancer incidence in Australian people living with HIV between 1982 and 2012. HIV Med. 2022;23(2):134‐145. doi:10.1111/hiv.13179 34585487 PMC10499845

[11] WHO Guidelines Review Committee . Consolidated guidelines on the use of antiretroviral drugs for treating and preventing HIV infection. 2016 update. 2016;155. 27466667

[12] Hoen B , Bonnet F , Delaugerre C , et al. French 2013 guidelines for antiretroviral therapy of HIV‐1 infection in adults. J Int AIDS Soc. 2014;17(1):19034. doi:10.7448/IAS.17.1.19034 24942364 PMC4062879

[13] Defossey G , Le Guyader‐Peyrou S , Uhry Z , et al. Estimations nationales de l'incidence et de la mortalité par cancer en France métropolitaine entre 1990 et 2018. Étude à Partir Des Registres Des Cancers du réseau Francim. 2019;1:372. http://www.santepubliquefrance.fr/

[14] Lanoy E , Spano JP , Bonnet F , et al. The spectrum of malignancies in HIV‐infected patients in 2006 in France: the ONCOVIH study. Int J Cancer. 2011;129(2):467‐475. doi:10.1002/ijc.25903 21207370

[15] Grabar S , Hleyhel M , Belot A , et al. Invasive cervical cancer in HIV‐infected women: risk and survival relative to those of the general population in France. Results from the French hospital database on HIV (FHDH)‐Agence Nationale de Recherches sur le SIDA et les Hepatites Virales (ANRS) CO4 cohort study. HIV Med. 2019;20(3):222‐229. doi:10.1111/hiv.12703 30693646

[16] Abraham AG , D'Souza G , Jing Y , et al. Invasive cervical cancer risk among HIV‐infected women: a north American multicohort collaboration prospective study. J Acquir Immune Defic Syndr. 2013;62(4):405‐413. doi:10.1097/QAI.0b013e31828177d7 23254153 PMC3633634

[17] McGee‐Avila JK , Argirion I , Engels EA , et al. Risk of hepatocellular carcinoma in people with HIV in the United States, 2001–2019. J Natl Cancer Inst. 2023;116:61‐68. doi:10.1093/jnci/djad172 PMC10777672 37610358

[18] Haas CB , Engels EA , Horner MJ , et al. Trends and risk of lung cancer among people living with HIV in the USA: a population‐based registry linkage study. Lancet HIV. 2022;9(10):e700‐e708. doi:10.1016/S2352-3018(22)00219-3 36179753 PMC9641618

[19] Shiels MS , Islam JY , Rosenberg PS , Hall HI , Jacobson E , Engels EA . Projected cancer incidence rates and burden of incident cancer cases in HIV‐infected adults in the United States through 2030. Ann Intern Med. 2018;168(12):866‐873. doi:10.7326/M17-2499 29801099 PMC6329294

[20] Engels EA , Shiels MS , Barnabas RV , et al. State of the science and future directions for research on HIV and cancer: summary of a joint workshop sponsored by IARC and NCI. Int J Cancer. 2024;154(4):596‐606. doi:10.1002/ijc.34727 37715370 PMC11133517

[21] Santé Publique France . Surveillance du VIH et des IST bactériennes en France en 2023. Bull Sante Publique. 2024;1‐35.

[22] Castilho JL , Bian A , Jenkins CA , et al. CD4/CD8 ratio and cancer risk among adults with HIV. J Natl Cancer Inst. 2022;114(6):854‐862. doi:10.1093/jnci/djac053 35292820 PMC9194634

[23] Chammartin F , Mocroft A , Egle A , et al. Measures of longitudinal immune dysfunction and risk of AIDS and non‐AIDS defining malignancies in antiretroviral‐treated people with human immunodeficiency virus. Clin Infect Dis. 2024;78(4):995‐1004. doi:10.1093/cid/ciad671 38092042 PMC11006099

[24] Sigel K , Dubrow R . CD4/CD8 ratio and lung cancer risk ‐ authors' reply. Lancet HIV. 2017;4(3):e103‐e104. doi:10.1016/S2352-3018(17)30026-7 28254147

[25] Severin D , Bessaoud F , Meftah N , et al. A comparative study of classic and HIV‐viremic and aviremic AIDS Kaposi sarcoma. Aids. 2021;35(3):399‐405. doi:10.1097/QAD.0000000000002744 33181532

[26] Palich R , Veyri M , Valantin MA , et al. Recurrence and occurrence of Kaposi's sarcoma in patients living with human immunodeficiency virus (HIV) and on antiretroviral therapy, despite suppressed HIV viremia. Clin Infect Dis. 2020;70(11):2435‐2438. doi:10.1093/cid/ciz762 31626689

[27] Guihot A , Dupin N , Marcelin AG , et al. Low T cell responses to human herpesvirus 8 in patients with AIDS‐related and classic Kaposi sarcoma. J Infect Dis. 2006;194(8):1078‐1088. doi:10.1086/507648 16991082

[28] Guiguet M , Boue F , Cadranel J , et al. Effect of immunodeficiency, HIV viral load, and antiretroviral therapy on the risk of individual malignancies (FHDH‐ANRS CO4): a prospective cohort study. Lancet Oncol. 2009;10(12):1152‐1159. doi:10.1016/S1470-2045(09)70282-7 19818686

[29] Caby F , Guiguet M , Weiss L , et al. CD4/CD8 ratio and the risk of Kaposi sarcoma or non‐Hodgkin lymphoma in the context of efficiently treated human immunodeficiency virus (HIV) infection: a collaborative analysis of 20 European cohort studies. Clin Infect Dis. 2021;73(1):50‐59. doi:10.1093/cid/ciaa1137 34370842

[30] Chalouni M , Wittkop L , Bani‐Sadr F , et al. Risk of severe clinical events after sustained virological response following direct‐acting antiviral therapy in HIV and hepatitis C virus coinfected participants. HIV Med. 2021;22(9):791‐804. doi:10.1111/hiv.13127 34212476

[31] Chalouni M , Pol S , Sogni P , et al. Increased mortality in HIV/HCV‐coinfected compared to HCV‐monoinfected patients in the DAA era due to non‐liver‐related death. J Hepatol. 2021;74(1):37‐47. doi:10.1016/j.jhep.2020.08.008 32798585

[32] van der Zee RP , Wit F , Richel O , et al. Effect of the introduction of screening for cancer precursor lesions on anal cancer incidence over time in people living with HIV: a nationwide cohort study. Lancet HIV. 2023;10(2):e97‐e106. doi:10.1016/S2352-3018(22)00368-X 36640800

[33] Clifford GM , Georges D , Shiels MS , et al. A meta‐analysis of anal cancer incidence by risk group: toward a unified anal cancer risk scale. Int J Cancer. 2021;148(1):38‐47. doi:10.1002/ijc.33185 32621759 PMC7689909

[34] Spindler L , Etienney I , Abramowitz L , et al. Screening for precancerous anal lesions linked to human papillomaviruses: French recommendations for clinical practice. Tech Coloproctol. 2024;28(1):23. doi:10.1007/s10151-023-02899-8 38198036 PMC10781838

