# Supplementary material for: Cancer risks and trends between 1997 and 2018, and effects of restored immunity in people living with HIV: Results from the ANRS CO4 French hospital database on HIV
Source: Int J Cancer. 2025 Nov 20;158(9):2298–308. doi: 10.1002/ijc.70253 (PMC12963702; doi:10.1002/ijc.70253)
Supplement: Supplementary file 1 — Appendix S1: Supporting information. [file IJC-158-2298-s001.pdf]

SUPPLEMENTARY MATERIAL

Cancer risks and trends between 1997-2018, and effects of restored immunity in people living with HIV.

Results from the ANRS CO4 French Hospital Database on HIV

Sophie Grabar, Paula Lakrout, Valérie Potard, Aurélien Belot, Jacques Cadranel, Christine Jacomet, Christine Katlama, Esaïe Marshall, Olivier Lambotte, Romain Palich, Sylvie Ronot, Jean-Philippe Spano, Anne-Marie Bouvier, Alain Makinson, Dominique Costagliola

TABLE OF CONTENTS:

SUPPLEMENTARY TABLES (p2-p6)

Supplementary Table 1: Prevalent and incident cancer case and person-year (p.y.) of follow-up by sex, period and cancer type. **p2**

Supplementary Table 2: ICD-10 codes of studied cancers. **p5**

Supplementary Table 3: Notification rates of cancers over 1997-2007 and 2008-2018 in ANRS CO4 FHDH. **p6**

SUPPLEMENTARY FIGURES (p7-p14)

Supplementary Figure 1: Flowchart. **p7**

Supplementary Figure 2: Age structure of the PWH population in 2013-2018 in ANRS CO4 FHDH and of the general population in 2013-2018 and 2008-2012 in France. **p8**

Supplementary Figure 3: Age standardized incidence rates and standardized incidence ratios (SIR) with 95% confidence intervals in PWH over 1997-2018 by group, with underreporting correction based on the notification rates estimated from the permanent beneficiary sample (EGB) for all periods. **p9**

Supplementary Figure 4: Age standardized incidence rates and standardized incidence ratios (SIR) with 95% confidence intervals in PWH over 1997-2018 by group, with underreporting correction based on ONCOVIH over 1997-2007 and on the notification rates estimated from the French 100% healthcare coverage program (HCP) from 2008. **p12**

SUPPLEMENTARY TABLES

Supplementary Table 1: Prevalent and incident cancer case and person-year (p.y.) of follow-up by sex, period and cancer type.

a/ Men

Sex	Period	Cancer	Number of prevalent cases	Number of participants in period	Number P.Y.	Number of incident cases (observed)	Number of incident cases (corrected)
Men	1997-2001	Kaposi's sarcoma	3 306	37 840	168 646	630	851
Men	2002-2007	Kaposi's sarcoma	3 936	46 019	268 530	623	841
Men	2008-2012	Kaposi's sarcoma	4 559	59 142	272 845	392	436
Men	2013-2018	Kaposi's sarcoma	4 951	72 068	365 885	341	379
Men	1997-2001	Non-Hodgkin lymphoma	1 847	39 299	175 897	557	785
Men	2002-2007	Non-Hodgkin lymphoma	2 404	47 551	278 976	542	763
Men	2008-2012	Non-Hodgkin lymphoma	2 946	60 755	282 104	367	427
Men	2013-2018	Non-Hodgkin lymphoma	3 313	73 706	376 807	356	414
Men	1997-2001	Hodgkin lymphoma	515	40 631	177 691	137	196
Men	2002-2007	Hodgkin lymphoma	652	49 303	281 876	182	260
Men	2008-2012	Hodgkin lymphoma	834	62 867	285 003	191	191
Men	2013-2018	Hodgkin lymphoma	1 025	75 994	380 343	174	174
Men	1997-2001	Anal cancer	2 317	38 829	178 209	62	172
Men	2002-2007	Anal cancer	2 379	47 576	282 729	155	431
Men	2008-2012	Anal cancer	2 534	61 167	286 064	102	309
Men	2013-2018	Anal cancer	2 636	74 383	381 803	154	467
Men	1997-2001	Liver cancer	525	40 621	178 274	57	83
Men	2002-2007	Liver cancer	582	49 373	283 132	165	239
Men	2008-2012	Liver cancer	747	62 954	286 521	182	350
Men	2013-2018	Liver cancer	929	76 090	382 468	220	326
Men	1997-2001	Lung cancer	779	40 367	178 200	124	218
Men	2002-2007	Lung cancer	903	49 052	283 021	216	379
Men	2008-2012	Lung cancer	1 119	62 582	286 399	241	298
Men	2013-2018	Lung cancer	1 360	75 659	382 205	398	491

Men	1997-2001	Colorectal cancer	342	40 804	178 222	31	135
Men	2002-2007	Colorectal cancer	373	49 582	283 050	65	283
Men	2008-2012	Colorectal cancer	438	63 263	286 434	85	370
Men	2013-2018	Colorectal cancer	808	76 211	382 138	170	739
Men	1997-2001	Prostate cancer	549	40 597	178 280	14	25
Men	2002-2007	Prostate cancer	563	49 392	283 033	97	170
Men	2008-2012	Prostate cancer	660	63 041	286 029	183	321
Men	2013-2018	Prostate cancer	843	76 176	380 850	346	607

b/ Women

Sex	Period	Cancer	Number of prevalent cases	Number of participants in period	Number P.Y.	Number of incident cases (observed)	Number of incident cases (corrected)
Women	1997-2001	Kaposi's sarcoma	574	16 176	72 995	61	82
Women	2002-2007	Kaposi's sarcoma	635	23 665	140 185	99	134
Women	2008-2012	Kaposi's sarcoma	734	32 858	149 212	53	59
Women	2013-2018	Kaposi's sarcoma	787	29 291	194 342	54	60
Women	1997-2001	Non-Hodgkin lymphoma	296	16 454	72 942	113	159
Women	2002-2007	Non-Hodgkin lymphoma	409	23 891	140 368	125	176
Women	2008-2012	Non-Hodgkin lymphoma	534	33 058	149 494	92	107
Women	2013-2018	Non-Hodgkin lymphoma	626	29 452	194 543	85	99
Women	1997-2001	Cervical cancer	120	16 630	72 513	57	75
Women	2002-2007	Cervical cancer	177	24 123	139 387	79	104
Women	2008-2012	Cervical cancer	256	33 336	147 186	71	91
Women	2013-2018	Cervical cancer	327	29 751	192 210	64	82
Women	1997-2001	Hodgkin lymphoma	51	16 699	73 286	20	29
Women	2002-2007	Hodgkin lymphoma	71	24 229	140 971	34	49
Women	2008-2012	Hodgkin lymphoma	105	33 487	150 144	36	36
Women	2013-2018	Hodgkin lymphoma	141	29 937	195 439	31	31
Women	1997-2001	Anal cancer	6	16 744	73 409	5	14

Women	2002-2007	Anal cancer	11	24 289	141 230	12	33
Women	2008-2012	Anal cancer	23	33 569	150 433	22	67
Women	2013-2018	Anal cancer	45	30 033	195 814	34	103
Women	1997-2001	Liver cancer	8	16 742	73 410	5	7
Women	2002-2007	Liver cancer	13	24 287	141 271	25	36
Women	2008-2012	Liver cancer	38	33 554	150 482	40	77
Women	2013-2018	Liver cancer	78	30 000	195 880	54	80
Women	1997-2001	Lung cancer	3	16 747	73 389	16	28
Women	2002-2007	Lung cancer	19	24 281	141 212	41	72
Women	2008-2012	Lung cancer	60	33 532	150 426	49	60
Women	2013-2018	Lung cancer	109	29 969	195 763	85	105
Women	1997-2001	Colorectal cancer	9	16 741	73 400	7	30
Women	2002-2007	Colorectal cancer	39	24 261	141 224	13	57
Women	2008-2012	Colorectal cancer	96	33 496	150 435	24	104
Women	2013-2018	Colorectal cancer	120	29 958	195 750	54	235
Women	1997-2001	Breast cancer	78	16 672	73 243	33	132
Women	2002-2007	Breast cancer	111	24 189	140 692	94	165
Women	2008-2012	Breast cancer	205	33 387	149 511	127	240
Women	2013-2018	Breast cancer	332	29 746	194 101	199	375

Notes:

Corrected number of observed cases: the number of observed cases were corrected according to the estimated notification rates of cancer reporting in the ANRS CO4 FHDH obtained in the ONCOVIH study (Lanoy et al. Int J Cancer 2011) for the period 1997-2007 and using a representative sample of the French health insurance beneficiaries for the period 2008-2018 (see **Supplementary Table 3**)

Supplementary Table 2: ICD-10 codes of studied cancers

		ANRS CO4 FHDH	FRANCIM
		**ICD-10 codes**	**ICD-Oncology 3**
Virus-related AIDS-defining cancers	Kaposi's Sarcoma	C46.x	C44.x and Morphology 9140
	Non-Hodgkin lymphoma	B211, B212, C833, C834, C837, C838, C839, C85, C851, C852, C857, C857+0, C859	Morphologies 9678-9684, 9687, 9688, 9712, 9735, 9737, 9738, 9826 whatever the topography
	Cervical cancer	C530, C531, C539	C53.x and Morphologies <95903
Virus-related non-AIDS-defining cancers	Hodgkin lymphoma	C81.x	Morphologies 9650-9655, 9659, 9661-9667 Whatever the topography
	Liver cancer	C22, C220-C224, C227, C229	C220 and Morphologies <95903
	Anal cancer	C211, C218 (sensitivity analysis with C210)	C21.x and Morphologies <95903
Virus-unrelated non-AIDS-defining cancers	Lung cancer	C33 and C34.x	C33.x-C44.x and Morphologies <95903
	Colorectal cancer	C18.x-C20.x	C18.x-C20.x and Morphologies <95903
	Prostate cancer	C61	C61 and Morphologies <95903
	Female breast cancer	C50.x	C50.x and Morphologies <95903

Abbreviations: AIDS, acquired immunodeficiency syndrome; ICD-10, International Classification of Diseases 10th Revision

<u>Supplementary Table 3</u>: Notification rates of cancers over 1997-2007 and 2008-2018 in ANRS CO4 FHDH

		Notification rates (%) ONCOVIH study 1997-2007	Notification rates (%) Total EGB 2008-2018	Notification rates (%) 100% HCP 2008-2018
Virus-related AIDS-defining cancers	Kaposi's sarcoma	0.74 [0.67-0.81]	0.90 [0.51-1.58]	>1
	Non-Hodgkin lymphoma	0.71 [0.67-0.75]	0.86 [0.50-1.49]	>1
	Cervical cancer	0.76 [0.57-1.00]	0.78 [0.19-3.13]	Not estimable
Virus-related non-AIDS-defining cancers	Hodgkin lymphoma	0.70 [0.63-0.79]	1.08 [0.45-2.62]	>1
	Liver cancer	0.69 [0.61-0.81]	0.52 [0.29-0.91]	>1
	Anal cancer	0.36 [0.29-0.48]	0.33 [0.19-0.57]	Not estimable
Virus-unrelated non-AIDS-defining cancers	Lung	0.57 [0.50-066]	0.81 [0.46-1.43]	>1]
	Colorectal cancer	*	0.23 [0.14-0.37]	0.38 [0.21-0.69]
	Prostate cancer	*	0.57 [0.32-1.01]	0.99 [0.47-2.08]
	Female breast cancer	0.25 [0.14-0.85]	0.53 [0.25-1.12]	0.62 [0.25-1.39]

EGB: Permanent beneficiary sample

Not estimable: no reported case in 100% HCP (Healthcare Coverage Program) for severe and costly long-term disease
100% HCP = French 100% Healthcare Coverage Program
ONCOVIH Study: published in Lanoy et al. Int J Cancer 2011

*Colorectal and prostate notification rates were not available in the Oncovih study therefore notification rates of total EGB 2008-2018 were applied for 1997-2007.

SUPPLEMENTARY FIGURES

Supplementary Figure 1: Flowchart

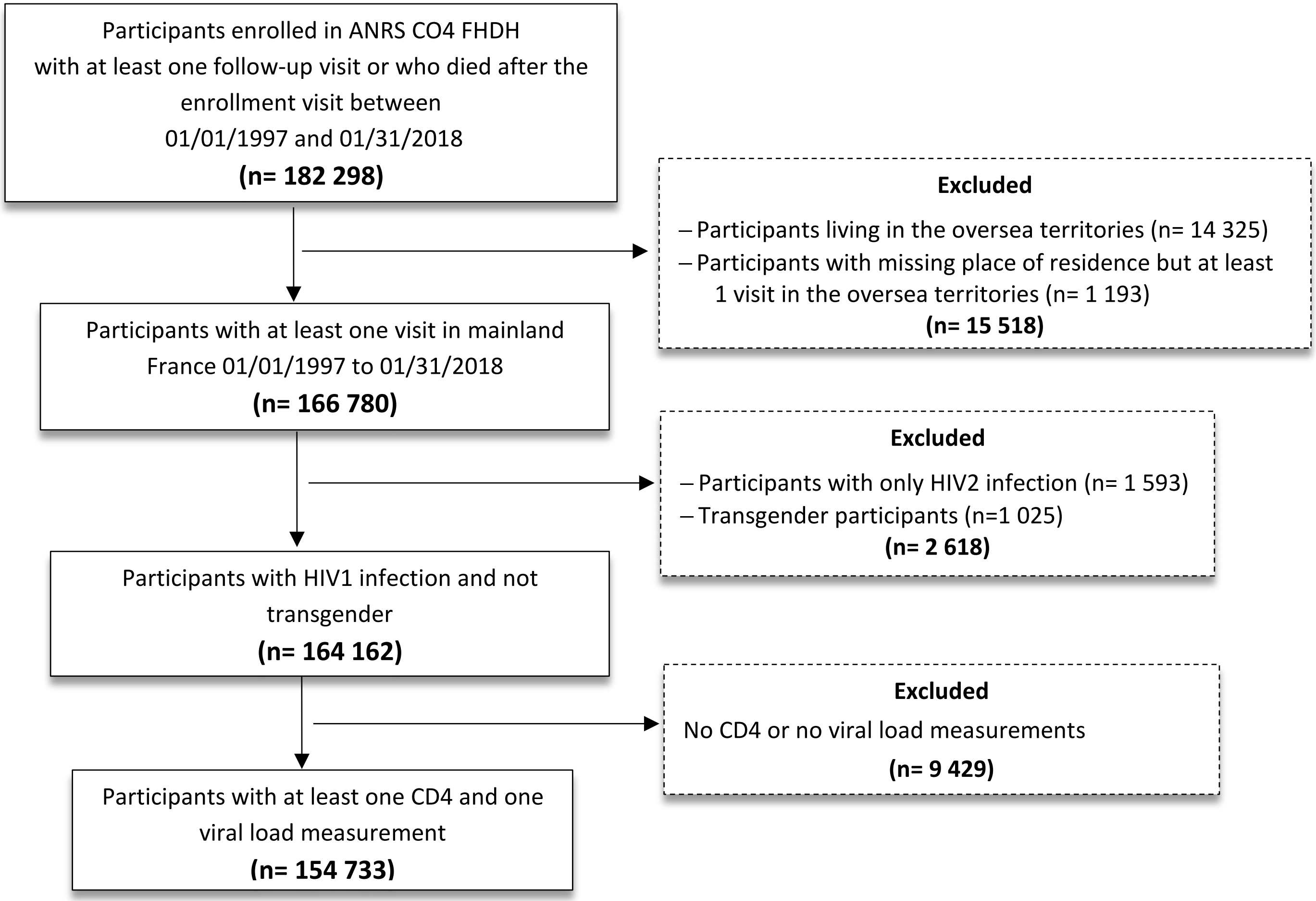

Supplementary Figure 2: Age structure of the PWH population in 2013-2018 in ANRS CO4 FHDH and of the general population in 2013-2018 and 2008-2012 in France.

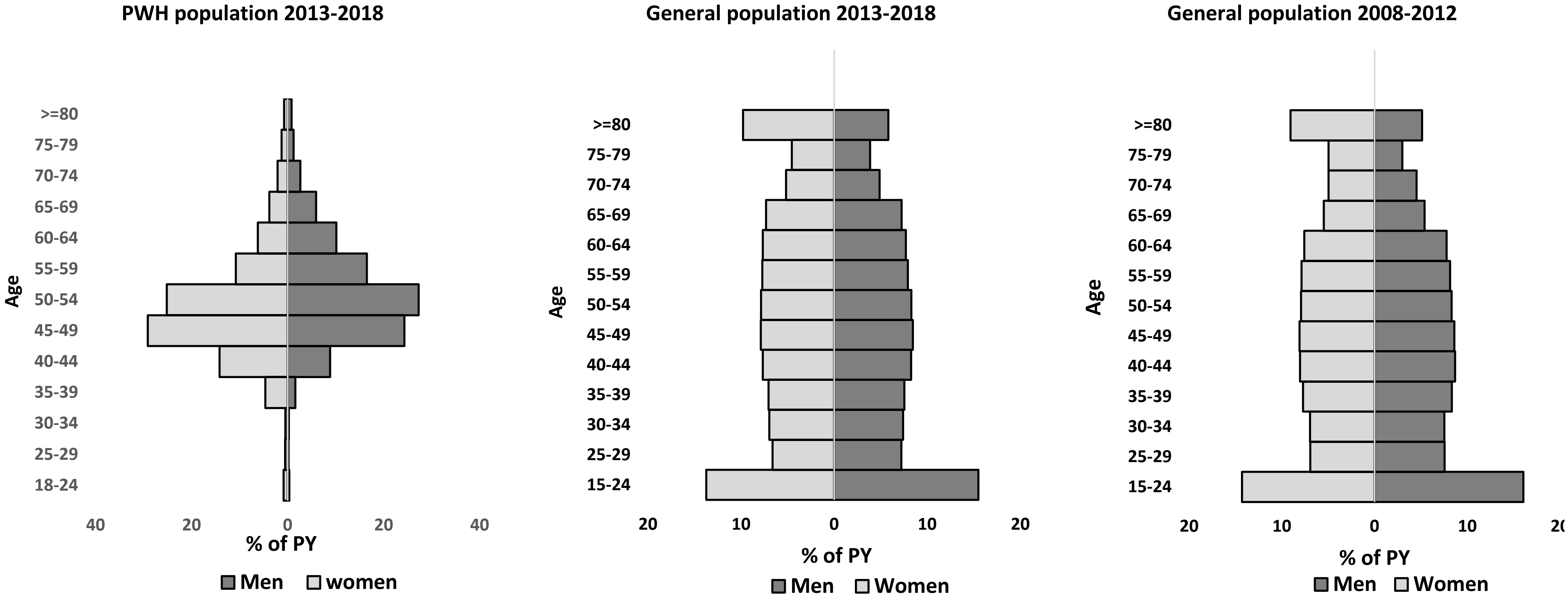

For the general population, person-years were calculated for the age defined as attained age, based on population data provided by the French National Institute of statistics and economic studies (Insee) updated in January 2020.

<u>Supplementary Figure 3</u>: Age standardized incidence rates and standardized incidence ratios (SIR) with 95% confidence intervals in PWH over 1997-2018 by group, with underreporting correction based on the notification rates estimated from the permanent beneficiary sample (EGB) for all periods.

<u>Supplementary 3.1</u>. Virus-related AIDS-defining cancers

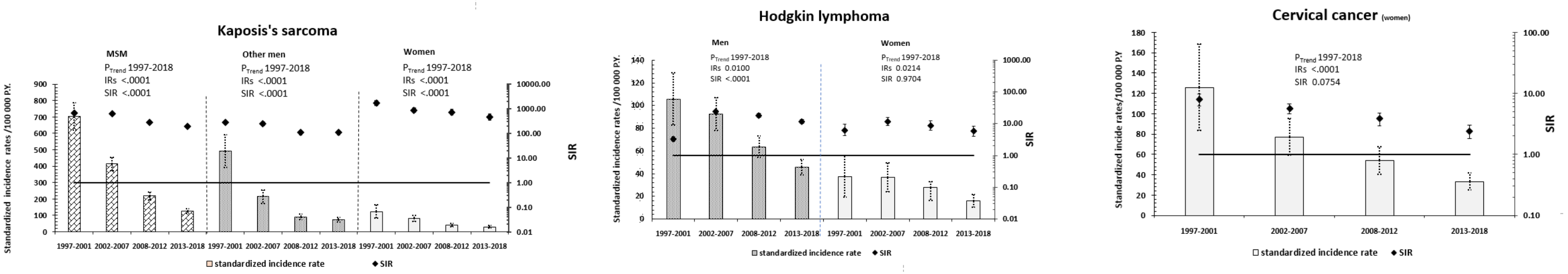

Supplementary 3.2. Virus-related non-AIDS-defining cancers

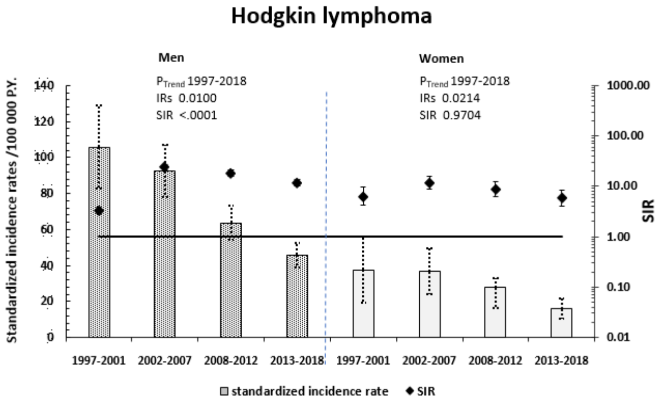

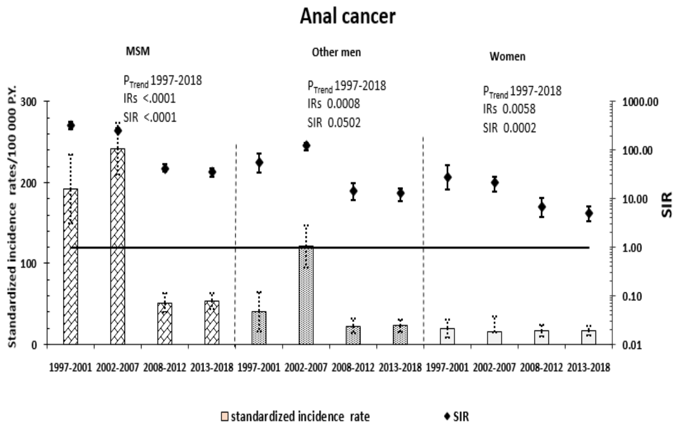

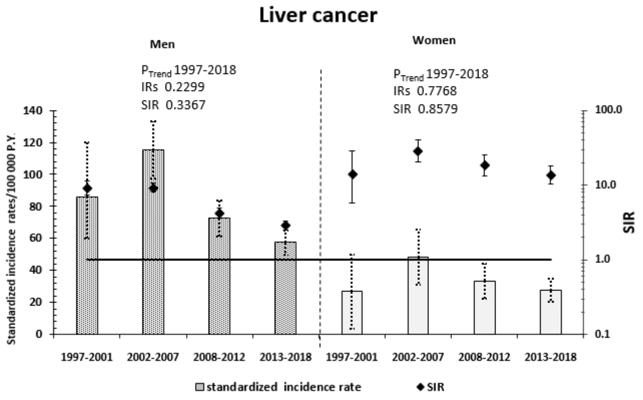

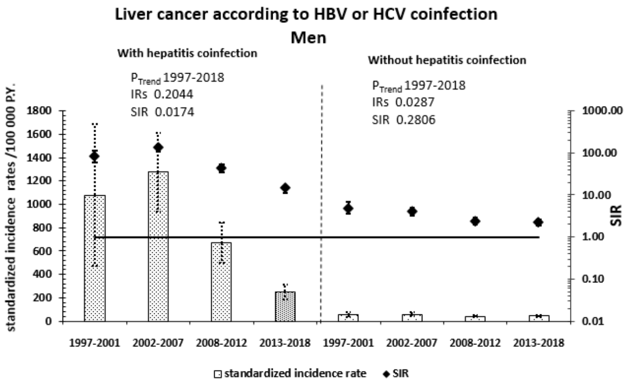

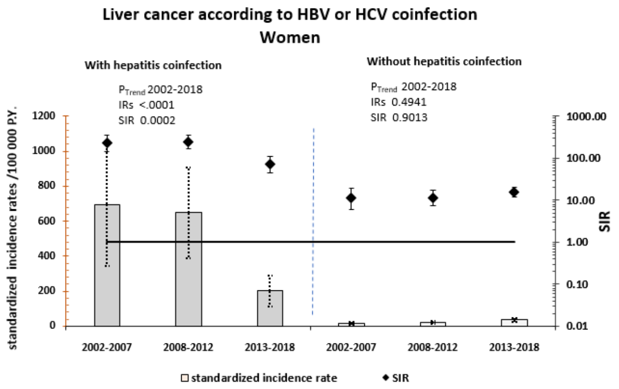

<u>Supplementary 3.3</u>. Virus-unrelated non-AIDS-defining cancers

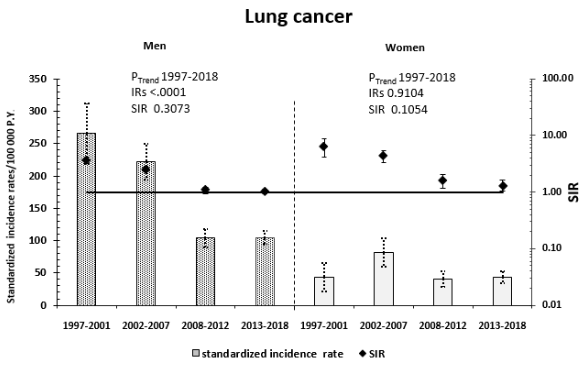

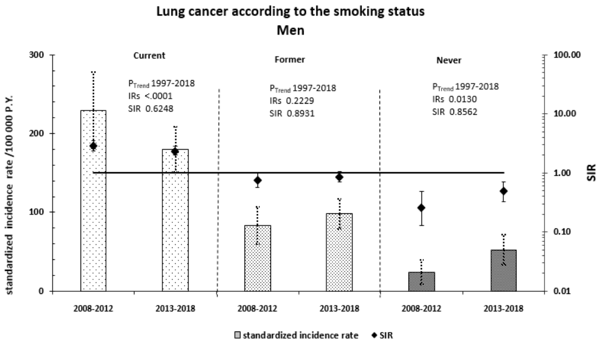

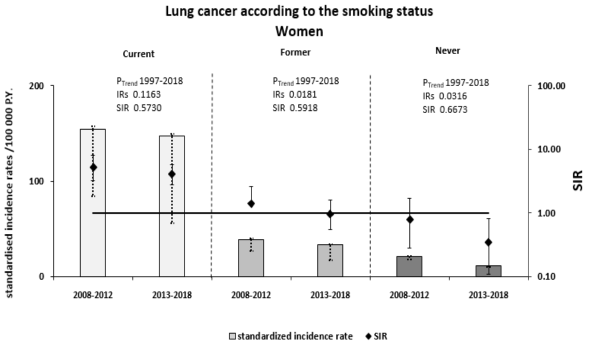

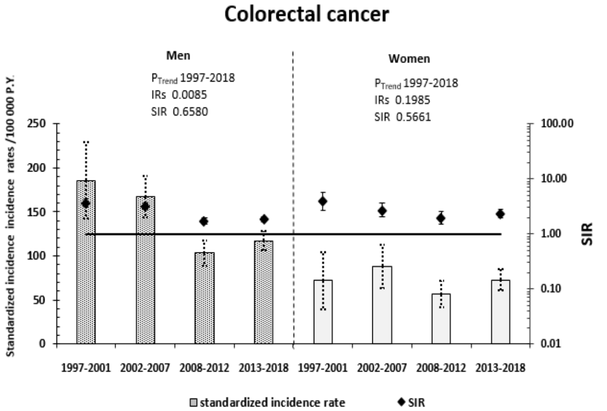

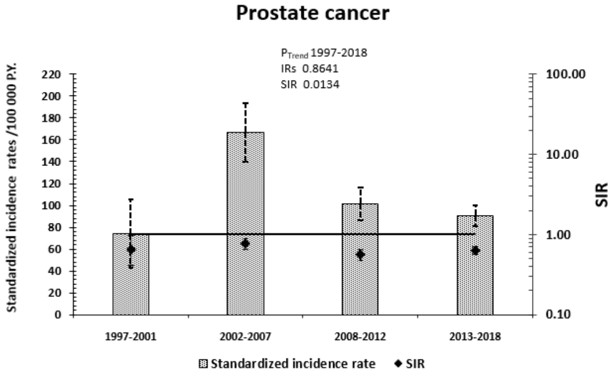

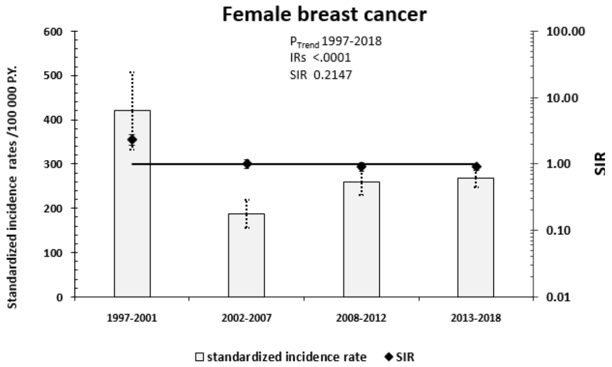

<u>Abbreviations</u>: SIR, standardized incidence ratio; PY, person-years; MSM, men who have sex with men

Incidence rates are standardized on the age distribution of PWH in FHDH between 2013-2018.
Standardized incidence ratio (SIR) versus the general population.
For liver cancer, the number of cases of HCV/HBV coinfected women, for the period 1997-2002 was too small to allow estimations.
Smoking was recorded after 2005 in FHDH and was missing for 42% of PWH followed since 2008.

<u>Supplementary Figure 4:</u> Age standardized incidence rates and standardized incidence ratios (SIR) with 95% confidence intervals in PWH over 1997-2018 by group, with underreporting correction based on ONCOVIH over 1997-2007 and on the notification rates estimated from the French 100% healthcare coverage program (HCP) from 2008.

<u>Supplementary 4.1.</u> Virus-related AIDS-defining cancers

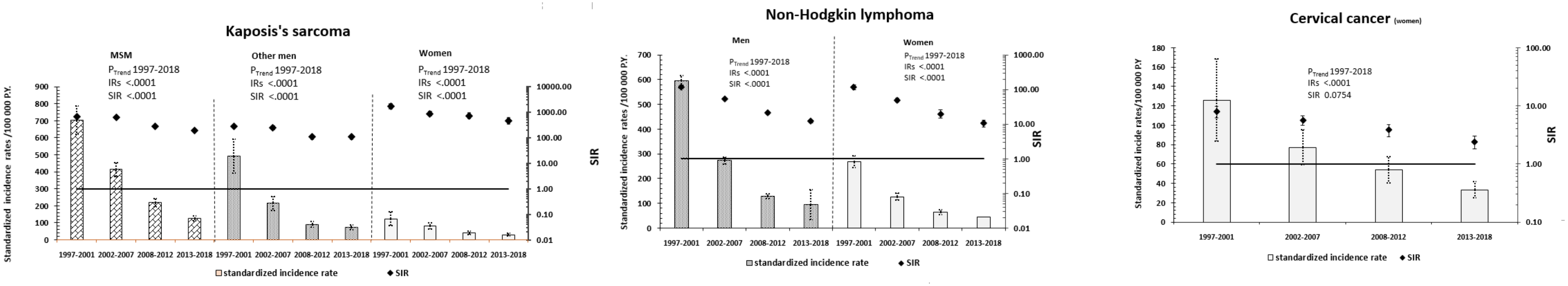

<u>Supplementary 4.2.</u> Virus-related non-AIDS-defining cancers

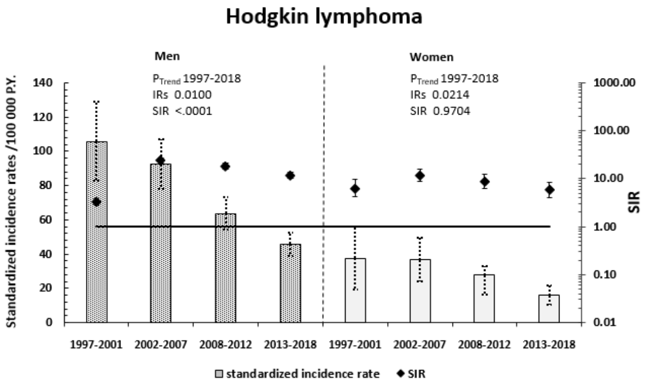

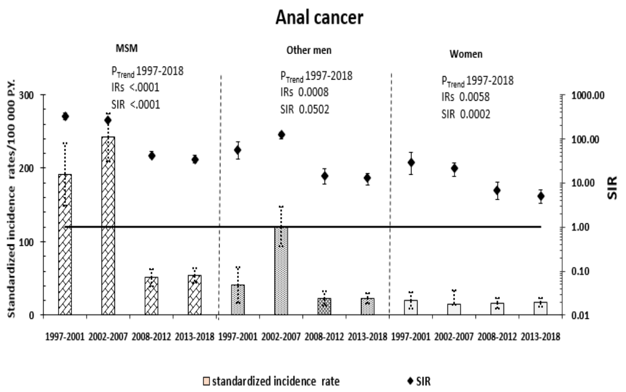

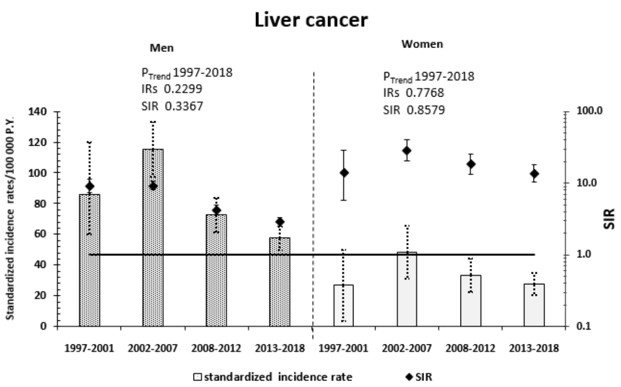

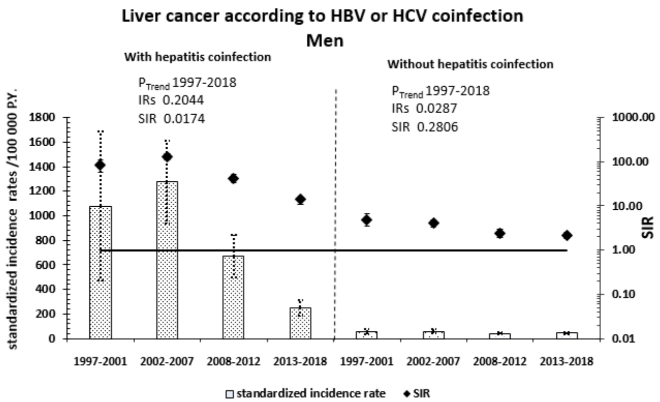

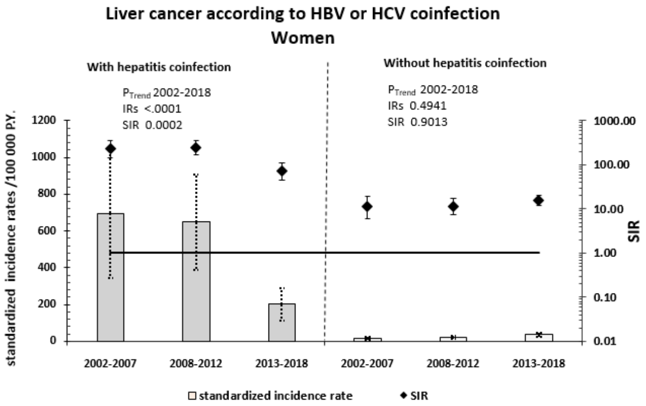

Supplementary 4.3. Virus-unrelated non-AIDS-defining cancers

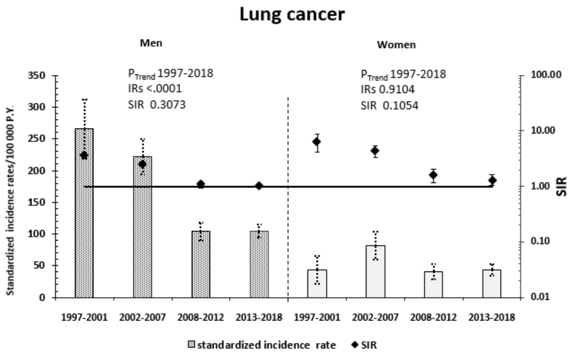

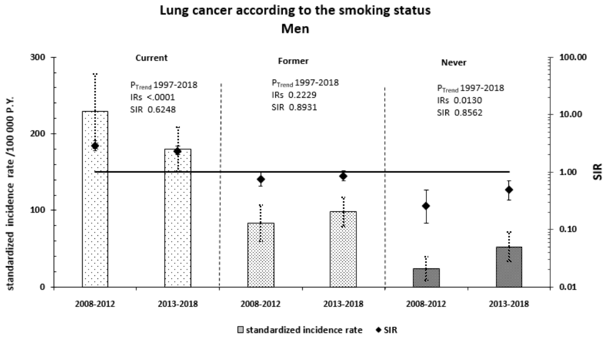

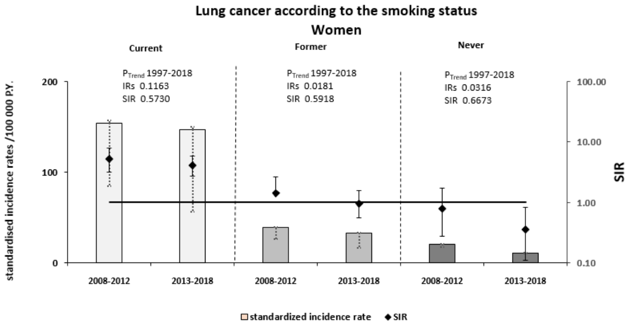

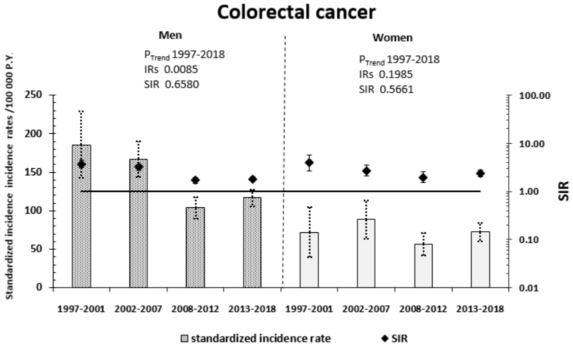

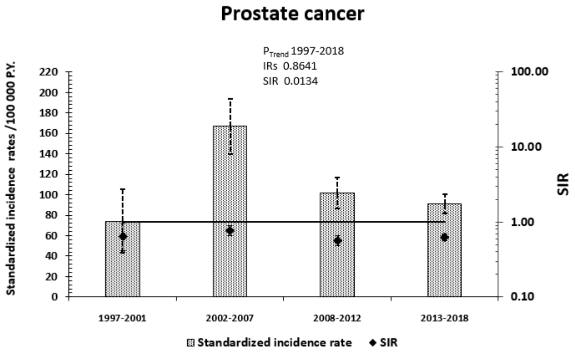

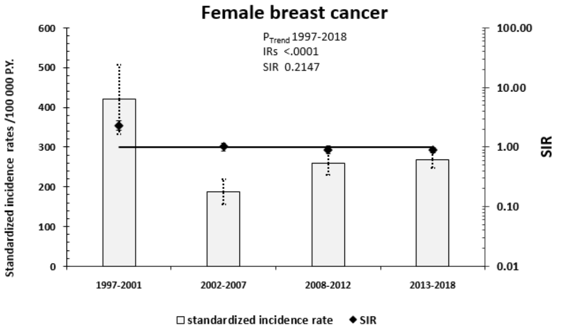

Abbreviations: SIR, standardized incidence ratio; PY, person-years; MSM, men who have sex with men

Incidence rates are standardized on the age distribution of PWH in FHDH between 2013-2018.
Standardized incidence ratio (SIR) versus the general population.
For liver cancer, the number of cases of HCV/HBV coinfected women, for the period 1997-2002 was too small to allow estimations.
Smoking was recorded after 2005 in FHDH and was missing for 42% of PWH followed since 2008.
